# Retraction: Low birth weight: causes and consequences

**DOI:** 10.1186/1758-5996-6-60

**Published:** 2014-05-27

**Authors:** Carlos Antonio Negrato, Marilia Brito Gomes

**Affiliations:** 1 Department of Internal Medicine, Bauru’s Diabetics Association, 17012-433 Bauru São Paulo, Brazil; 2 Department of Internal Medicine, Diabetes Unit, State University Hospital of Rio de Janeiro, Rio de Janeiro, Brazil

## 

This article [1] has been retracted by the Editor due to extensive overlap with a number of different previously published articles.
